# Improved Methods for Assessing Therapeutic Potential of Antifungal Agents against Dermatophytes and Their Application in the Development of NP213, a Novel Onychomycosis Therapy Candidate

**DOI:** 10.1128/AAC.02117-18

**Published:** 2019-04-25

**Authors:** Derry K. Mercer, Colin S. Stewart, Lorna Miller, Jennifer Robertson, Vanessa M. S. Duncan, Deborah A. O’Neil

**Affiliations:** a NovaBiotics, Ltd., Craibstone, Aberdeen, United Kingdom

**Keywords:** antifungal agents, antifungal susceptibility testing, antimicrobial peptides, dermatophytes, onychomycosis, tinea

## Abstract

Onychomycosis is a common, difficult-to-treat nail infection that is mainly caused by dermatophytes. Current therapies are not wholly effective and are associated with manifold side effects. The development of treatments for onychomycosis is challenging because standard *in vitro* tests are not predictive of antifungal efficacy within the nail. We have developed a new antifungal agent, NP213, for the treatment of onychomycosis. NP213 is based on endogenous host defense peptides produced within the nail. We compared the *in vitro* activity of NP213 and existing antifungal agents using conventional antimicrobial susceptibility test (AST) systems and more physiologically relevant models based on the human nail. We observed that the standard *in vitro* AST methodologies failed to predict the efficacy of antifungal agents within the nail. To address that, we present a more physiologically relevant modified AST method. This method, alongside other standard *in vitro* assessments of activity (including mechanism-of-action and time-of-kill studies), better reflected the activity of NP213 and other antifungal agents within the nail than standard *in vitro* AST methods. NP213 is a rapidly acting, fungicidal peptide that is superior to existing antifungal agents *in vitro*. It penetrated the nail more effectively than other antifungals, as confirmed by using an optimized *in vitro* nail infection model. The data presented here support the current clinical development status of NP213 as a novel agent for treating onychomycosis. We propose that the modified tests developed and applied for NP213 characterization are the most relevant to use for screening any potential therapeutic candidates for onychomycosis.

## Abstract (precis)

Onychomycosis is a common, difficult-to-treat nail infection that is mainly caused by dermatophytes. Current therapies are not wholly effective and are associated with manifold side effects.

## INTRODUCTION

Onychomycosis (fungal infection of the nail) is notoriously difficult to treat. The majority of patients receiving any of the limited number of currently available treatments (mainly azole or allylamine based) often fail to respond or relapse (1–3). The nail is a highly effective biological barrier and, hence, delivery of therapeutic agents to the nail and nail bed is challenging (4, 5). Keratin, the major constituent of the nail and the primary carbon and nitrogen source for dermatophytes, binds to and inactivates many of the existing small molecule antifungal classes, further compromising therapy (6–8). Not surprisingly perhaps, the overall efficacy of current antifungal agents in onychomycosis is poor (4). Paradoxically, the potent *in vitro* antifungal data for the active agents of these therapies would indicate otherwise. On the one hand, safer, more effective therapies are required for onychomycosis. On the other hand, more clinically predictive, physiologically relevant *in vitro* systems to test new compounds with therapeutic potential for this common condition are required.

To circumvent the limitations and challenges of drug delivery to the nail, we have taken a biological approach to combatting onychomycosis and designed a novel therapeutic agent derived from antimicrobial, or host defense, peptides (HDPs). HDPs are an essential component of the innate immune response to infection and are expressed and produced in the skin and nail (9–12). We have developed a synthetic antimicrobial peptide, NP213 (Novexatin), which is now in late-stage clinical development as a topical therapy (in a water-based solution) for the treatment of onychomycosis. Preliminary data from a phase I and IIa clinical study in a total of 60 subjects confirmed that when dosed daily for 28 days, NP213 was very well tolerated in patients with onychomycosis and brought about improvement of nail appearance (during and sustained after application; the NP213 effect was greater than that of a placebo) and mycological improvement/resolution of infection when nails were assessed for dermatophyte burden 180 days after a 28-day application cycle of NP213 or placebo (the NP213 effect was greater than placebo in patients with mild to moderate onychomycosis) (unpublished data). Here, we present the development and characterization of NP213 using standard *in vitro* tests, as well as a novel *in vitro* nail infection system and a modified *in vitro* MIC determination protocol developed here as more physiologically relevant for testing the activity of antidermatophyte agents. We show that NP213 is a promising, novel, first-in-class therapy candidate for nail fungus. We also demonstrate a reverse paradox to that detailed above for small molecule antifungals. Namely, standard *in vitro* antimicrobial susceptibility test (AST) systems (e.g., Clinical and Laboratory Standards Institute [CLSI] or European Committee on Antimicrobial Susceptibility Testing [EUCAST] methods employing nutrient-rich media [13–15]) do not accurately predict the activity of NP213 and other antifungals within the nail. We recommend use of the optimized *in vitro* nail infection system and modified *in vitro* AST protocol we present here as a novel development strategy, in conjunction with standard *in vitro* tests, for more accurate assessment of the curative potential of new onychomycosis agents.

## RESULTS AND DISCUSSION

### Rational design of NP213.

Most HDPs are short (<50 amino acids [aa]), cationic, and amphipathic peptides produced by all complex life forms as first line defense against microbes (16–18). Using HDP as a blueprint, NP213 (Arg_7_) was designed as a highly hydrophilic and positively charged (net charge +7) cyclic peptide. We anticipated that the charge would facilitate nail penetration by NP213, since the nail is a negatively charged aqueous hydrogel under physiological conditions (19). Further, NP213 is smaller than endogenous HDPs (7 aa versus ca. 30 < 100 aa) that are known to be present within the nail (9, 11, 12, 20, 21). One of the known drawbacks of peptide and protein therapeutic candidates is their susceptibility to hydrolysis, especially proteolysis (22). This is of particular concern with antidermatophyte agents, since dermatophytes produce multiple proteases and peptidases that enable them to hydrolyze keratin (23–25). NP213 was designed specifically to circumvent these issues. First, as an all-arginine peptide, the limited sequence diversity and only R-R bonds within the peptide are susceptible to very limited classes of endoproteases (26). Second, the cyclic structure of NP213 minimizes the probability of hydrolysis by exoproteases. NP213 was synthesized by standard solid-phase synthesis processes as an acetate peptide salt in amorphous crystalline form, its purity determined by reversed phase-high performance liquid chromatography (data not shown), and used as an aqueous solution for the ensuing *in vitro* testing.

### *In vitro* activity of NP213 against dermatophytes using conventional test methods.

**FIG 1:**
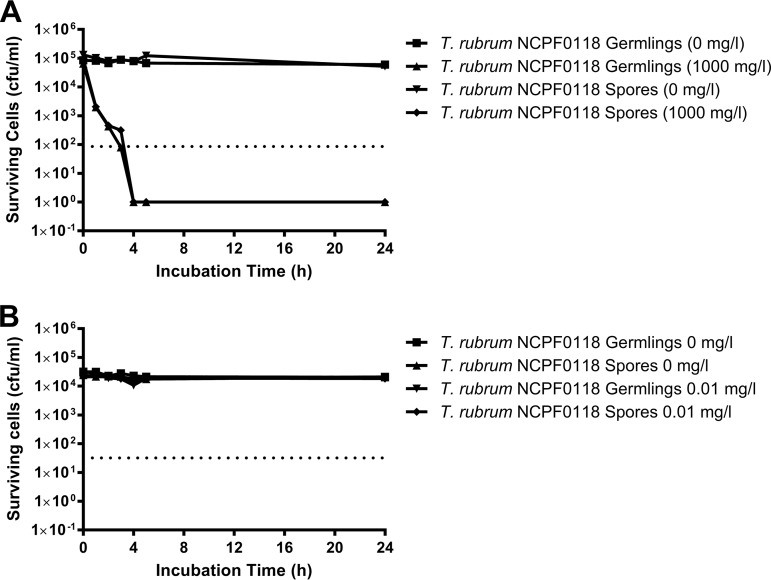
Incubation time required for fungicidal activity of NP213 or terbinafine (TRB) against spores or germlings (germinated hyphae) of T. rubrum NCPF0118 and T. rubrum ATCC MYA-4438. (A) Antimicrobial efficacy of NP213 against T. rubrum NCPF0118. (B) Antimicrobial efficacy of TRB against T. rubrum NCPF0118. Experiments were conducted three times with three technical replicates in each experiment. The data shown are from one representative experiment. Dotted lines on the graphs represent 3 log (99.9%) kill.

**(i) Time-of-kill, sporicidal, and antihyphal activity.** Dermatophytes grow vegetatively as hyphae but also produce spores (micro/macroconidia and occasionally chlamydospores), which are dormant, asexual, and nonmotile cells and are normally less susceptible to the antifungal activity of existing drug classes, including terbinafine (TRB), ciclopirox, amorolfine, and bifonazole, than actively metabolizing cells (27–30). Spore production in the nail (31) is thought to contribute to treatment failure and recurrence of onychomycosis (32). The time of kill of a nominal dose of 1,000 mg/liter NP213 was therefore determined against both spores and hyphae of T. rubrum NPCF0118. NP213 was equally efficacious against spores and hyphae of T. rubrum NCPF0118; a >3-log (99.9%) kill of spores and hyphae of T. rubrum NCPF0118 took 3 to 4 h (6.47 × 10^4^ to 9.47 × 10^4^ CFU/ml killed). In contrast, 0.01 mg/liter (2× MIC) of TRB failed to kill spores or hyphae of T. rubrum NCPF0118, even after 24 h (Fig. 1). This demonstrates that NP213 is fungicidal and killed more rapidly than TRB (33) under these test conditions.

**FIG 2:**
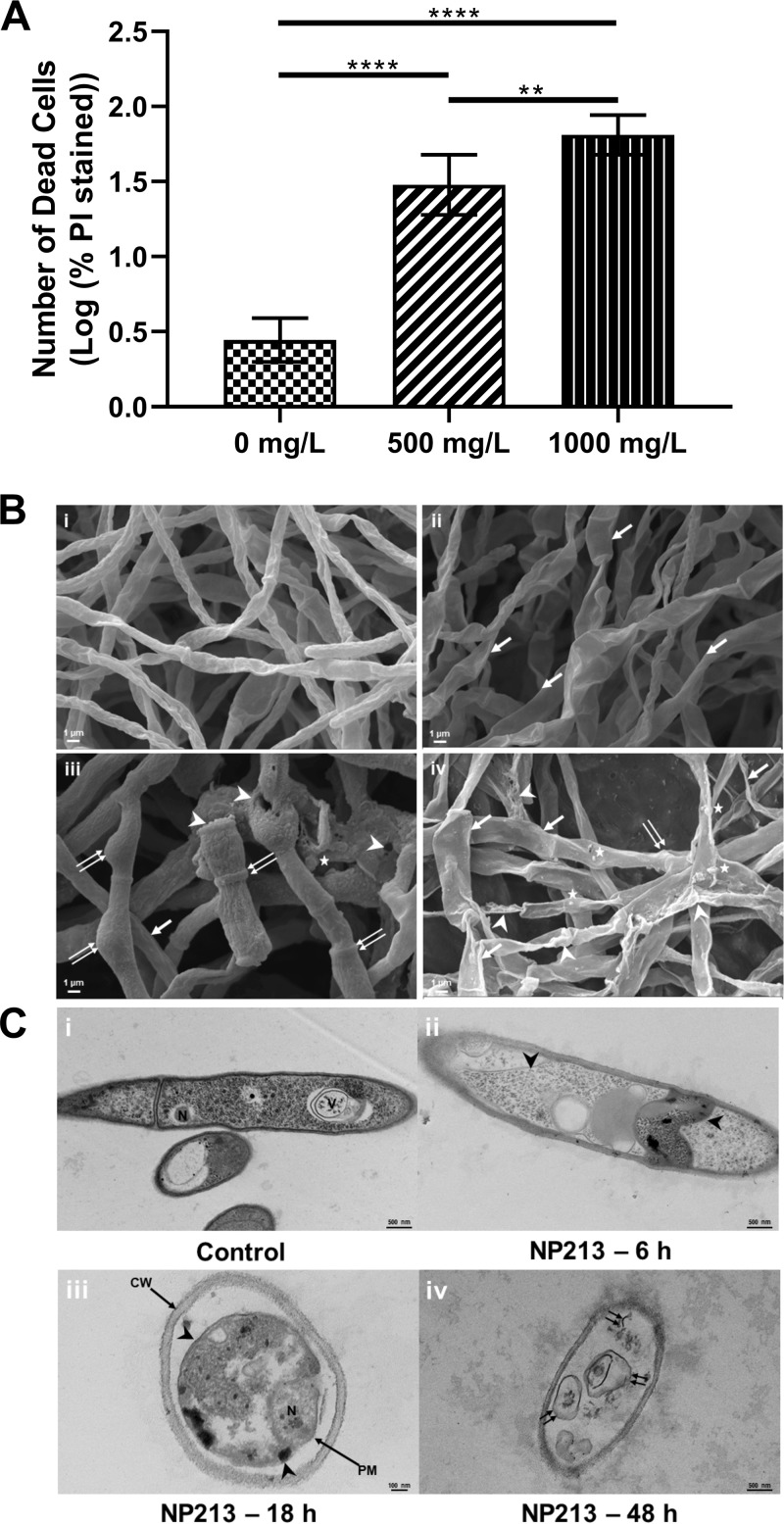
Mode of action of NP213. (A) Propidium iodide (PI) staining of T. rubrum NCPF0118 incubated with NP213. T. rubrum NCPF0118 spores were exposed to NP213 for 18 h at 30°C, followed by a 15-min exposure to PI. Cells were examined by fluorescence microscopy (× 400 magnification). The total number of cells and the number of PI-stained cells were counted in six independent fields of view for each sample in duplicate experiments. The log-transformed mean number of PI-stained cells is presented, and error bars represent 95% confidence intervals. Statistical analysis was conducted using one-way ANOVA, followed by Tukey’s multiple-comparison test to determine significance (****, *P* < 0.0001; **, *P* < 0.01) between NP213-treated and untreated cell populations. (B) SEM analysis of the effect of a 48-h exposure to antifungals on the morphology of T. rubrum NCPF0118. (i) Untreated cells; (ii) 100 mg/liter ciclopirox; (iii) 0.5 mg/liter TRB; (iv) 100 mg/ml NP213. Single arrows, flattening of the hyphae; double arrows, bulges within the hyphae; arrowheads, damage to the surface of the hyphae; asterisks, possible blebbing on the hyphal surface. All sample treatments were conducted in triplicate, and at least 30 images were acquired for each treatment. The figures are representative images from a single replicate. (C) TEM analysis of the effect of duration of incubation with NP213 (10 mg/ml) on the morphology of T. rubrum NCPF0118. CW, cell wall; PM, plasma membrane; N, nucleus; V, vacuole. Arrowheads, separation of the plasma membrane from the cell wall; double arrows, plasma membrane destruction. The images are representative of three replicates. Scale bars represent 500 nm for the control, 6-h, and 48-h treatments and 100 nm for the 18-h treatment.

**(ii) NP213 targets the fungal cytoplasmic membrane.** We hypothesized that the antifungal activity of NP213 occurs via membrane perturbation and disruption/permeabilization, since that is the most common mechanism of action of the cationic HDP used as a blueprint for the design of NP213 (18, 34). To establish whether that this was indeed the case, propidium iodide (PI) staining of a spore suspension of T. rubrum NCPF0118 exposed to 500 or 1,000 mg/liter NP213 for 18 h was performed. PI is a membrane-impermeant fluorescent dye that fluoresces when it intercalates double-stranded DNA (35). Therefore, it is a useful indicator of membrane permeabilization and cell death (Fig. 2A). The number of PI-stained T. rubrum NCPF0118 cells in samples incubated with NP213 was significantly higher than in controls in the absence of NP213 (*P* < 0.005; one-way analysis of variance [ANOVA] with Tukey’s multiple-comparison test following log transformation of data) and was dose dependent since the difference in the number of PI-stained cells was significantly higher when T. rubrum NCPF0118 was exposed to 1,000 mg/liter NP213 compared to exposure to 500 mg/liter NP213 (*P* < 0.01; one-way ANOVA with Tukey’s multiple-comparison test following log transformation of data). This suggested that membrane permeabilization occurred upon exposure to a range of concentrations of NP213. This further confirmed that NP213 was fungicidal and that at least one of its mechanisms of action involved membrane permeabilization.

Scanning electron microscopy (SEM) and transmission electron microscopy (TEM) analyses were then performed to investigate the effect of NP213 on T. rubrum cell morphology directly. For SEM analysis, T. rubrum NCPF0118 cells were exposed to NP213 (100× MIC, 100 g/liter), and two marketed antifungal agents, TRB (0.5 mg/liter, 100× MIC) or ciclopirox olamine (100 mg/liter, 100× MIC), for 48 h before SEM analysis (Fig. 2B and Fig. S1). The appearance of cells exposed to ciclopirox and TRB was similar to that of untreated cells. In contrast, the hyphae of cells exposed to NP213 appeared flatter, which was indicative of a potential loss of internal turgor pressure following membrane lysis (Fig. 2Biv). The presence of cellular debris on the surface of hyphae supported the occurrence of cell lysis. Since the SEM analysis cannot be used for a direct evaluation of membrane lysis, the effect of NP213 on T. rubrum NCPF0118 was also analyzed by TEM (Fig. 2C). For TEM analysis, cross sections of hyphae exposed to 10 g/liter NP213 (10× MIC) for 0, 6, 18 or 48 h revealed clear loss of cellular contents (Fig. 2Cii to iv) compared to control cells, in which distinct cellular organelles were visible (Fig. 2Ci). This indicated that unlike currently available onychomycosis agents, NP213 is novel in directly targeting the fungal cytoplasmic membrane. Further, NP213 significantly reduced the number of germ tubes formed by T. rubrum NCPF0118 (Fig. S2), although it did not affect the length of the formed germ tubes (data not shown).

**FIG 3:**
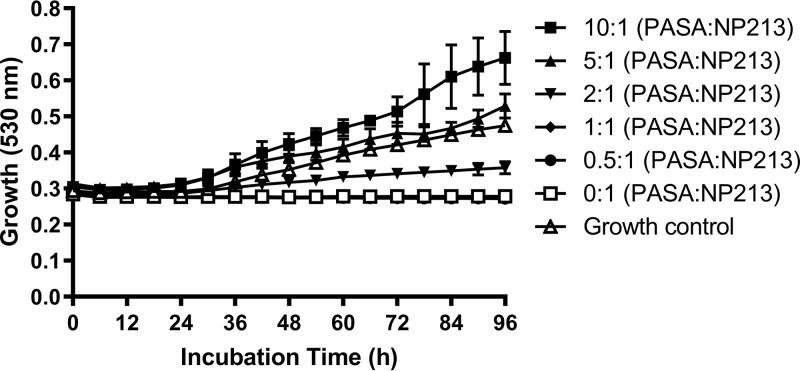
Antifungal activity of NP213 is dependent on its positive charge. T. rubrum NPCF0118 (ca. 1 × 10^3^ CFU/ml) was incubated aerobically for 96 h at 30°C in the presence of 1 mM NP213 (1,093.3 mg/liter) and increasing molar equivalents of PASA in RPMI 1640 liquid medium. The data are presented as means, and error bars represent the 95% confidence intervals of three biological replicates, each containing three technical replicates. Data were analyzed for significance by two-way ANOVA, with Dunnett’s multiple-comparison test. PASA alone, at the concentrations used, had no effect on the growth of T. rubrum NCPF0118 (data not shown).

**(iii) Antimicrobial activity of NP213 is cationic charge dependent.** Each molecule of NP213 has a net positive charge of +7 to facilitate penetration of the nail (19–21) and to enable initial binding of NP213 to negatively charged microbial membranes via electrostatic interactions, a characteristic of most positively charged HDPs (18, 36, 37). The importance of the positive charge on the antifungal activity of NP213 was investigated by incubating T. rubrum NCPF0118 with 1 mM NP213 and different molar equivalents of polyanetholesulfonic acid (PASA; Liquoid) (Fig. 3). PASA is an anionic derivative of polystyrene that binds to and neutralizes antimicrobial factors in the blood, including complement (38). As the concentration of PASA increased relative to that of NP213, exceeding the equimolar ratio, the inhibition of growth of T. rubrum NCPF0118 by NP213 became less evident. The data were analyzed by two-way ANOVA and Dunnett’s multiple-comparison test to compare the effect of PASA with no exposure to PASA, which indicated that, once the molar ratio of PASA to NP213 exceeded 2:1, then the lack of growth inhibition due to neutralization of the positive charge of NP213 was statistically significant (*P* < 0.0001). Thus, the positive charge of NP213 was indeed essential for its antifungal activity.

**(iv) NP213 MIC values determined using standard broth microdilution AST.** Considering its effect on fungal growth *in vitro*, the MICs of NP213 required to achieve these actions were determined using the current standard broth microdilution procedure for filamentous fungi (13) against a broad range of dermatophytes and nondermatophyte fungi that cause onychomycosis. The dermatophyte MICs ranged from <100 to 4,000 mg/liter, and the MFC values ranged from 500 to 4,000 mg/liter (a complete MIC data set for dermatophytes is provided in Table S1 in the supplemental material). NP213 also demonstrated activity against yeasts and nondermatophyte fungi known to cause onychomycosis (data not shown). The dermatophyte MIC values, while high compared to values obtained for other antifungals, including TRB, ciclopirox, and azoles used to treat onychomycosis, nevertheless demonstrated that NP213 can inhibit growth of a range of dermatophytes causative of onychomycosis. The possibility of adaptive resistance to NP213 by selected target dermatophytes was assessed *in vitro*, with no evidence of resistance development against NP213 over 20 passages but with evidence of resistance to TRB (data not shown).

It is now widely recognized that standard antimicrobial susceptibility testing protocols, including CLSI (13) and EUCAST (15) protocols, fail to adequately account for the environmental factors that are present during host-pathogen interactions *in vivo* and can appreciably impact antibiotic efficacy *in vitro* and that the media on which these tests are based do not mimic the host environment (39–43). Furthermore, standard antimicrobial susceptibility tests were devised as long ago as 1961 and were not designed to interrogate the activity of nonantibiotic antimicrobials, including antimicrobial peptides such as NP213. We therefore developed and tested novel *in vitro* methods for antimicrobial susceptibility testing of antidermatophyte agents under more physiologically appropriate conditions.

### *In vitro* activity and *ex vivo* efficacy of NP213 testing using novel, physiologically relevant methods.

**(i) Development of a modified MIC determination method for dermatophytes.** The broth microdilution methods used to determine the MIC and MFC values utilize the relatively nutrient-rich RPMI 1640 liquid medium as a test matrix (13). This medium contains 2 g/liter glucose as the main carbon source, no protein, and only small amounts of individual amino acids (0.01 to 0.2 g/liter). In contrast, the human nail is predominantly made up of different types of keratin that are highly cross-linked by disulfide bonds (44, 45). Consequently, the nutrient content of RPMI 1640 liquid medium bears little resemblance to the nutrients available to fungi growing in or on the nail.

**FIG 4:**
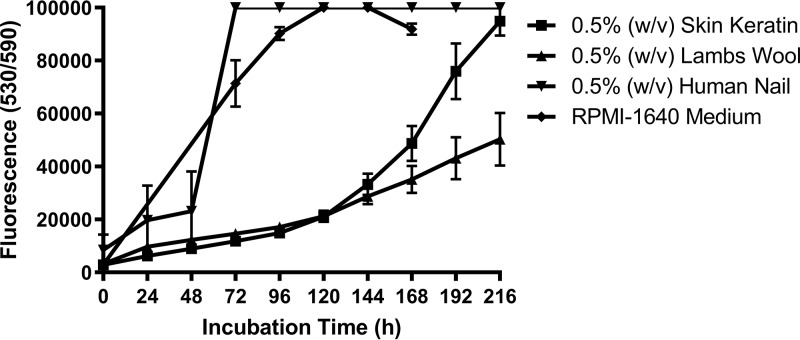
Effect of keratin source on the metabolic activity of T. rubrum NCPF0118. T. rubrum NCPF0118 (ca. 1 × 10^3^ spores/ml) was inoculated into 10 mM sodium phosphate buffer (pH 7.0) containing 0.5% (wt/vol) of each keratin source. Cultures were incubated for up to 216 h at 30°C and metabolic activity (alamarBlue reduction) was monitored every 24 h. Experiments were conducted three times with three technical replicates per experiment, and data are presented as means ± the standard deviations.

We sought to develop an antifungal susceptibility testing protocol that would resemble clinical and/or *in vivo* conditions more closely than conventional MIC testing methods, based on keratin, the major carbon and nitrogen source for dermatophytes within human nail. The medium used for broth microdilution MIC testing (13) was therefore modified to a protein-based test medium containing almost no carbohydrate (44) from the carbohydrate-rich (2.0% [wt/vol] glucose), protein-free RPMI 1640 liquid medium. Different sources of keratin, representative of keratin-rich epithelia and known to support dermatophyte growth, were selected (46–49). Powdered human nail material, human skin keratin, or sheep’s wool keratin, all used as suspensions in 10 mM sodium phosphate buffer (pH 7.0), were investigated as a nutrient source *in vitro* for dermatophyte growth. Suspensions of test media precluded monitoring fungal growth by turbidity, and so antifungal susceptibility was assessed using fluorescence-based determination of fungal metabolism using the metabolic activity indicator, alamarBlue (50). In preliminary antimicrobial susceptibility testing in modified medium containing a nominal concentration of the different keratins at 0.5% (wt/vol), the MIC values of NP213 against T. rubrum NCPF0118 were 4- to 62.5-fold lower than those obtained when the fungi were grown in RPMI 1640 liquid medium (Table 1). Interestingly, MICs varied, depending on the source of the keratin, with MICs for NP213 lowest in media containing powdered human nail. To determine whether these reductions in MIC, particularly that seen in human nail powder-based media, was in part due to reduced background metabolism of the fungi versus when in glucose-rich RPMI 1640, the metabolic growth of T. rubrum NCPF0118 was assessed for up to 168 h in 10 mM sodium phosphate buffer (pH 7.0) containing 0.5% (wt/vol) concentrations of the different keratins was assessed without the presence of NP213. This revealed (Fig. 4) that in fact only fungi cultured in the human nail powder-derived keratin had a metabolic growth profile in line with that achieved in RPMI 1640. Growth was somewhat reduced versus either of these culture conditions in the skin and sheep’s wool keratin-based media. Human nail material was therefore selected for subsequent studies since not only is this the most relevant keratin source when investigating onychomycosis and potential therapeutic agents but seemed to best support the growth of T. rubrum. We next determined whether 0.5% (wt/vol) nail powder was optimal as the concentration of nutrient source to employ in these assays. Interestingly, concentrations as low as 0.1% (wt/vol) human keratin supported the metabolic activity of T. rubrum NCPF0118, but not to the extent where metabolic activity was in line with that seen in RPMI 1640 liquid medium as a benchmark, whereas concentrations in the range of 0.25 to 0.75% (wt/vol) nail powder (plateauing thereafter) in 10 mM sodium phosphate did achieve the same metabolic activity as RPMI 1640 liquid medium and were employed in subsequent experiments.

**TABLE 1:** Effect of keratin source on the antifungal activity of NP213 against T. rubrum NCPF0118^*a*^

Keratin source (0.5% [wt/vol]) in phosphate buffer^*b*^	Median MIC (mg/liter)
Human nail	16–32
Human skin	125
Lamb’s wool	250
RPMI 1640	1,000

a In these experiments, the MIC was defined as the concentration of NP213 required for a 100% inhibition of fungal metabolic activity, determined by the modified version of the broth microdilution procedure (see Materials and Methods for complete details). Median values are shown for three technical replicates from three replicate experiments.

b Phosphate buffer refers to 10 mM sodium phosphate buffer (pH 7.0).

**(ii) Antifungal activity of NP213 assessed using the modified MIC determination method.** The MIC values of NP213 were further investigated in the modified antimicrobial test system (containing only human nail material) alongside those for TRB and ciclopirox olamine against T. rubrum NCPF0118. Activity in this system was compared to MICs obtained using the CLSI broth microdilution protocol (Table 2). The MIC (determined by the CLSI broth microdilution procedure) of NP213 was reduced by up to 128-fold when determined using the modified protocol. The apparent MIC of TRB increased 2- to 16-fold and that of ciclopirox increased as much as 16-fold but was not completely inhibited despite it being described that these antifungal agents are known to bind to nail keratin with a concomitant inhibition of antifungal efficacy (6, 8, 51). We believe that this nail keratin-based medium (containing 0.5% [wt/vol] powdered human nail) is optimal for determining the antifungal activity of a range of onychomycosis treatment candidates since it supports high levels of dermatophyte metabolic activity (Fig. 4), while at the same time being a more accurate demonstration of the physiological conditions encountered by onychomycosis-causing fungi in the nail.

**TABLE 2:** Antifungal activity of NP213, terbinafine hydrochloride, and ciclopirox olamine was determined against T. rubrum NCPF0118 in RPMI 1640 liquid medium or 10 mM sodium phosphate buffer (pH 7.0), supplemented with powdered human nail material^*a*^

T. rubrum NCPF0118	MIC_100_ in mg/liter (MIC fold change^*b*^)		
	NP213	Terbinafine	Ciclopirox
T. rubrum in RPMI 1640	1,000	0.005	0.5
T. rubrum in buffer with nail content (% [wt/vol])			
0.1	8–32 (↓32–128)	0.01–0.02 (↑2–4)	2–4 (↑4–8)
0.25	8–32 (↓32–128)	0.01–0.04 (↑2–8)	2–4 (↑4–8)
0.5	16–32 (↓32–64)	0.01–0.08 (↑2–16)	2–4 (↑4–8)
0.75	8–32 (↓32–128)	0.02–0.04 (↑4–8)	8 (↑16)

a MIC_100_ values were determined using a modified broth microdilution procedure for incubation with human nail material and a standard broth microdilution in RPMI 1640 (13) and then compared. The MICs reported for RPMI 1640 medium are determined based on optical density changes at 530 nm and metabolic activity, with identical results. All experiments were conducted at least three times, with three technical replicates for each experiment.

b The MIC fold change in human nail compared to the MIC obtained in RPMI 1640 liquid medium is indicated in parentheses.

Overall, our data support the application of the modified MIC method we developed in the assessment of activity of antifungal agents against dermatophytes. The levels of keratin in this medium are not inhibitory to antifungal agents known to bind to and be inactivated by keratin. This method facilitates testing against fungi in a metabolic state more closely resembling their status within the nail and as detailed below, activity of these agents within the nail.

**FIG 5:**
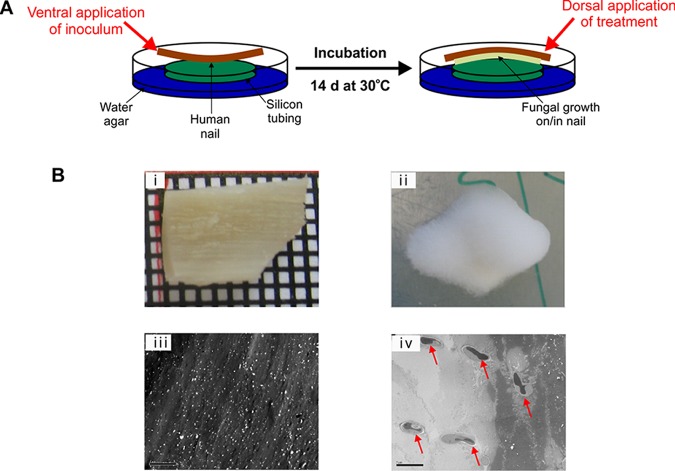
Optimized *in vitro* human nail model for evaluating the efficiency of antidermatophyte agents. (A) Diagram of the *in vitro* human nail model of *Trichophyton* infection. Sterile nails were inoculated with *Trichophyton* spp. (ca. 2 × 10^7^ spores/ml) on the ventral (lower) side and incubated at 30°C for 14 days, at which point the nail surface was entirely covered by hyphal growth, and fungi had penetrated into the nail matrix. The nail fragments were then inverted so that the dorsal (upper) surface was facing upward. Investigative substances and controls were applied on the dorsal side only. (Bi to iv) Images of an uninfected and infected nail from the *in vitro* nail infection model. (i) Sterile nail fragment prior to inoculation with T. rubrum NCPF0118; (ii) nail fragment following infection with T. rubrum NCPF0118 for 14 days at 30°C; (iii) TEM of medial section of a sterile human nail fragment; (iv) TEM of medial section of a human nail fragment following infection with T. rubrum NCPF0118 for 14 days at 30°C. Arrows indicate cross-sectional views of fungal hyphae within the nail matrix. The images are representative of three replicates. Scale bars, 5 μm.

**FIG 6:**
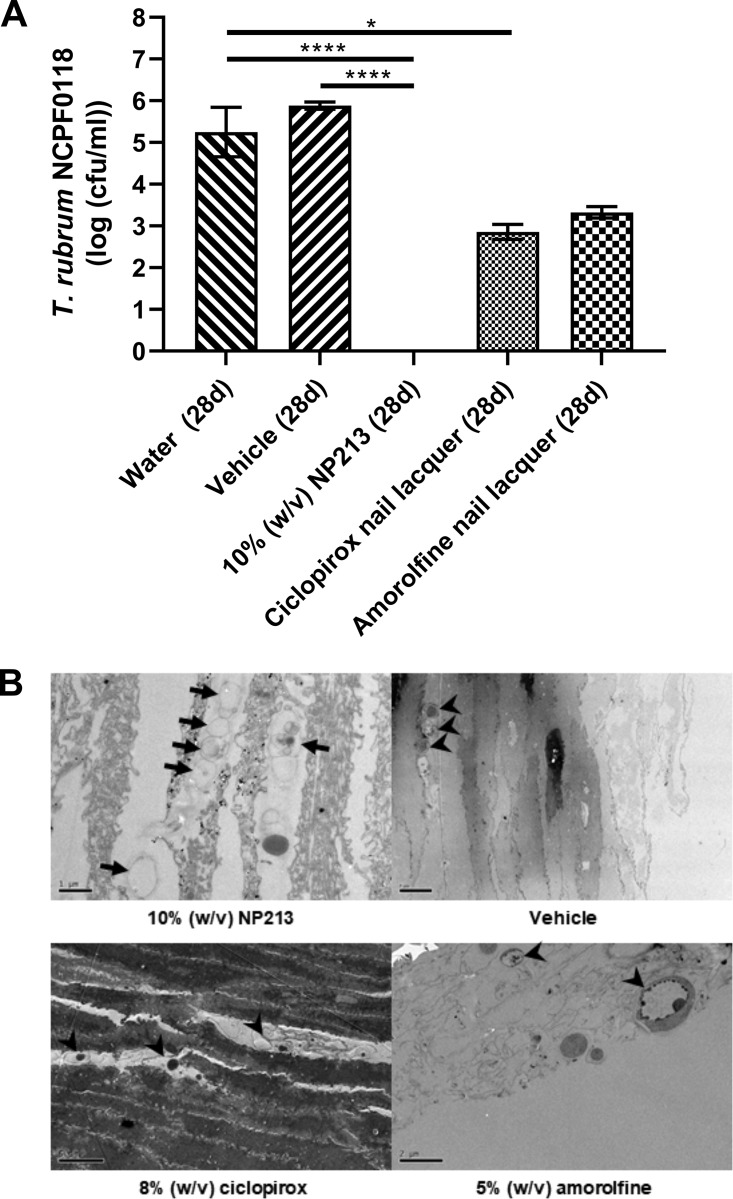
Efficacy of NP213 and other antifungal agents in the *in vitro* model of infection. (A) Antimicrobial activity of an NP213 (in a water-based vehicle), 8% (wt/vol) ciclopirox nail lacquer, and 5% (wt/vol) amorolfine nail lacquer against T. rubrum NCPF0118. The data are representative of triplicate samples from three nail fragments and are presented as the log-transformed mean; error bars represent the 95% confidence intervals. Statistical analysis was conducted using Kruskal-Wallis test of log-transformed CFU/ml, followed by Dunn’s multiple-comparison test to determine significance (****, *P* < 0.0001; *, *P* < 0.05). (B) TEM analysis of the effect of NP213, ciclopirox, and amorolfine on fungal morphology within the nail after treatment for 28 days with 10% (wt/vol) NP213, 8% (wt/vol) ciclopirox nail lacquer, or 5% (wt/vol) amorolfine nail lacquer against T. rubrum NCPF0118. Nails were treated on a daily basis with the described compounds (and incubated at 30°C) and processed after 28 days to count the live fungi (colony counting) and for TEM. For the TEM analysis, arrows indicate probable dead fungi with cell walls remaining but no evidence of intracellular content. Arrowheads indicate probable live fungi with evidence of intracellular content. The images are representative of three replicate nail fragments per treatment. Scale bars: 1 μm (NP213), 2 μm (amorolfine), 5 μm (ciclopirox and vehicle).

**(iii) Optimization of an *in vitro* model of nail infection.** The *in vitro* activity and mechanism of action determined for NP213, the MIC data obtained using the modified MIC method, and preliminary clinical data obtained thus far together suggest that NP213 might be highly efficacious in the nail environment. *In vitro* nail models have previously been described for testing candidate onychomycosis therapies, including those based on sections of human nail, animal hoof slices, and films of hair keratin (52–55). However, these are not entirely physiologically relevant because animal hooves do not closely resemble the human nail in terms of keratin content. Further, the human nail models generally do not use full-thickness human nails or only use very small fragments thereof. We therefore optimized the *in vitro* full-thickness model of dermatophyte nail infection (as described in Materials and Methods; see also Fig. 5). A higher fungal inoculum in our own model versus other published *in vitro* models (2 × 10^7^ spores/nail fragment) facilitated successful establishment of deep-seated infections with T. rubrum NCPF0118 (Fig. 5B and 6B). Similar infections were also established with T. rubrum ATCC MYA-4438 and T. rubrum S52d0 (data not shown).

**(iv) Antifungal activity of NP213 in the optimized *in vitro* model of nail infection.** We next used the *in vitro* nail model to determine whether NP213 was active against *Trichophyton* spp. infecting the human nail. Excised human nails were prepared and infected as described in Materials and Methods. NP213 (in a water-based vehicle) or comparator currently marketed antifungals were applied to infected nails for 28 days on a daily basis. Viable fungi were extracted and cultured on potato dextrose (PDA) agar to assess the infection status of the nails, as detailed in Materials and Methods. NP213 application to nails successfully eradicated different strains of T. rubrum from infected nails after only 28 days of daily application compared to vehicle alone or water (Table 3), unlike the comparator topical onychomycosis agents ciclopirox and amorolfine (Fig. 6A). Importantly, there was no evidence of a placebo effect for NP213, as treatment with the vehicle alone caused no significant reduction in the number of T. rubrum CFU recovered at the end of the experiments (Kruksal-Wallis test of log-transformed data [*P* < 0.0001]) (Table 3 and Fig. 6A). Placebo formulations are not available for the ciclopirox or amorolfine comparator products, meaning that a placebo effect of these organic-solvent containing products cannot be ruled out as a contributing factor of any antifungal effects observed for these products. In addition to culturing viable dermatophytes, fungal elements within the nail were analyzed by TEM (Fig. 6B), revealing only the presence of nonviable hyphal “ghosts” (loss of intracellular contents from fungi, but fungal cell walls remaining relatively intact) in NP213-treated nails and more evidence of cellular damage than the intact, viable cells within infected nails treated with ciclopirox or amorolfine (or other agents, such as itraconazole [data not shown]). Importantly, these data also confirm NP213’s ability to penetrate full thickness nail and remain bioactive therein, since the sections visualized by TEM were those were taken from the ventral aspect of nail samples.

**TABLE 3:** Survival of T. rubrum in *in vitro*-infected human nail model^*a*^

T. rubrum strain	Dermatophytes recovered (log mean CFU/ml [log 95% CI])			*P* (NP213:vehicle)
	Prior to treatment	Vehicle	10% (wt/vol) NP213	
NCPF0118	6.6 (6.5–6.7)	5.9 (5.5–6.3)	0.0 (0.0, 0.0)	0.0082
ATCC MYA-4438	4.3 (4.0–4.7)	6.0 (5.7–6.5)	0.0 (0.0, 0.0)	<0.0001
S29d0	6.5 (6.2–6.8)	7.0 (6.7–7.2)	0.0 (0.0, 0.0)	<0.0001

a The survival of T. rubrum in the *in vitro* infected human nail model (14 days at 30°C) after exposure to 10% (wt/vol) NP213 in a water-based vehicle or control vehicle without NP213 following daily application for 28 days. Data are presented as the log-transformed means and 95% confidence intervals (CI) and analyzed by using a Kruskal-Wallis test. Data are derived from three nail fragments per treatment and triplicate counts per nail fragment.

**FIG 7:**
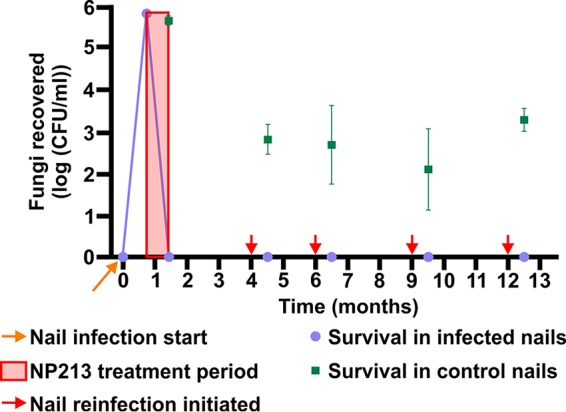
NP213 remains active within the human nail for at least 335 d posttreatment termination, as evidenced by a lack of T. rubrum NCPF0118 growth following attempted reinfection of previously treated nail fragments. Fungal recovery from the control nails infected 28, 120, 180, 270, and 365 days after the initial infection and from nails originally infected with T. rubrum NCPF0118 and treated with 10% (wt/vol) NP213 daily for 28 days at 30°C was determined as described in Materials and Methods. After the treatment phase, nails were incubated at 30°C for the time points specified, in a humid atmosphere until processing to determine the numbers of surviving fungi. Log-transformed colony counts represent the means and 95% confidence intervals of triplicate samples from each of the three nails per time point.

The activity/biostability of NP213 within full-thickness human nail was further assessed for up to 11 months following cessation of application of NP213 for 28 days to eradicate an *in vitro* nail infection (Fig. 7). Infected and NP213-treated nails, cleared of fungal infection, were reexposed to T. rubrum NCPF0118 after 3, 5, 8, and 11 months following the cessation of NP213 treatment. Infection could not be reestablished in NP213-treated nails but was successfully established in control, untreated nails inoculated alongside as controls (Fig. 7). Log-transformed data for each reinfection time point were analyzed by one-way ANOVA with Holm-Sidak’s multiple-comparison test to compare vehicle and NP213 treatment, and the reduction in counts following NP213 treatment was statistically significant (*P* < 0.0001) at every reinfection time point.

**FIG 8:**
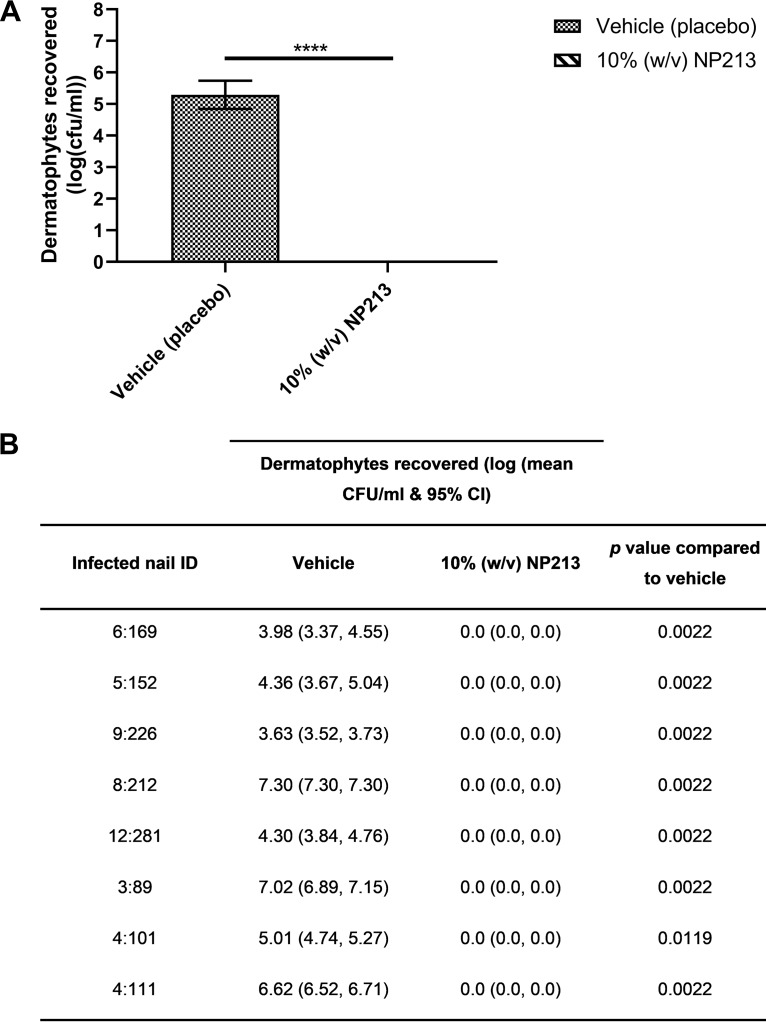
*Ex vivo* efficacy of NP213 against onychomycotic nails. Eight great (large) toe nails from patients with clinically confirmed onychomycosis were treated daily with 10% (wt/vol) NP213 in an aqueous water-based vehicle or vehicle only for 28 days before recovery of dermatophytes (triplicate counts per nail fragment) on DTM agar. (A) Combination data representing the activity of NP213 on all samples compared to vehicle (unpaired *t* test with Welch’s correction following log transformation of data; *P* < 0.0001). (B) Table showing the effect on individual nails. Treatment of nails with NP213 caused a statistically significant reduction in the number of CFU recovered for all nail samples treated with NP213 compared to vehicle (Mann-Whitney test following log transformation of data).

To further validate our *in vitro* nail model and, importantly, to confirm that NP213 was also able to eradicate infection from full-thickness, onychomycotic nails from patients with established, chronic clinical infection, we assessed NP213 efficacy *ex vivo* in infected nails from patients with clinically confirmed onychomycosis caused by dermatophytes (Fig. 8). Only dermatophytes were isolated and enumerated from untreated nails, facilitated by the use of selective dermatophyte test medium (DTM) (Becton Dickinson UK, Ltd), but it is possible that other infectious agents were present in the naturally infected nails. Moreover, nails may have been infected with more than one dermatophyte strain. Nonetheless, NP213 successfully eradicated all dermatophyte infection from these onychomycotic nails with dermatophyte burdens of 4.3 × 10^3^ CFU/ml to >2 × 10^7^ CFU/ml, a “natural state” inoculum range replicated in our *in vitro* nail model described above.

The predicted value of the novel, combined testing strategy developed here, combining the standard and modified *in vitro* assays and *in vitro* nail model assays is, we believe, significant for future testing of novel antidermatophyte agents. Alongside standard *in vitro* tests, the approach we now recommend mitigates, in part, the paradox of *in vitro* testing results and activity within the nail for antidermatophyte agents. It also provides conditions that are more representative of the behavior of dermatophytes within the nail and of the behavior of antifungal agents in a nutrient-poor biological matrix such as the nail.

### Conclusions.

The need for new, safe, and more effective antifungal agents as onychomycosis therapies is obvious and significant. Further, a clear need exists for more predictive *in vitro* testing methods that can be applied to all antifungal agents. We addressed the first point, the need for new antifungals, by developing NP213. We addressed the second point, the need for better testing methods, by developing a novel, expanded testing system that goes beyond standard, non-physiologically relevant MIC experiments and employs nail infection models and human nail-based media (the latter as the basis for modified AST assays). Importantly, the tests described here are predictive of the activity of antifungal agents within the nail and allow them to be tested against pathogenic dermatophytes in a (metabolic) state in which they would exist within the nail. Without this full package of data, the high MIC/MFC values obtained using standard methods (Table 1) would likely have been considered too high to pursue further development of NP213, a now late-clinical-stage therapeutic candidate for onychomycosis.

## MATERIALS AND METHODS

### Peptides and chemicals.

NP213 was synthesized as an acetate salt (>95% purity) by solid-phase synthesis (PolyPeptide Group, France; Almac Group, UK; Ambiopharm, Inc.). NP213 was prepared as a lyophilized powder and dissolved in sterile-deionized water (sdH_2_O) to a concentration of 400 mg/ml, unless otherwise stated. All other chemical and growth media were obtained from Sigma-Aldrich (UK), except where otherwise stated.

### Fungal isolates.

A complete list of fungal isolates used in these experiments can be found in Table S1. Dermatophytes were purchased from the National Collection of Pathogenic Fungi (Public Health England, UK), the American Type Culture Collection (LGC Standards, UK), and the Deutsche Sammlung von Mikroorganismen und Zellkulturen GmBH (DSMZ, Germany). Clinical isolates of dermatophytes were obtained from Michel Monod (Centre Hospitalier Universitaire Vaudois, Switzerland) and Boni Elewski (University of Alabama at Birmingham). Dermatophyte cultures were grown on Sabouraud dextrose (SAB) agar (Oxoid, Ltd., UK), for up to 14 days at 30°C. Fungi were stored at –85°C with 0.5% (vol/vol) dimethyl sulfoxide as cryoprotectant. For experimental purposes, dermatophytes were cultured at 30°C for up to 14 days on PDA (Oxoid, Ltd).

### Time-of-kill method.

Spore suspensions of ∼1 × 10^5^ spores/ml were prepared (13) and used directly to determine the time-of-kill for spores. To test the germlings (germinated spores/short hyphae), spores were allowed to germinate for 12 h at 30°C, examined microscopically (Axiovert 40 CF microscope; Carl Zeiss, Ltd., UK) to ensure that germination had occurred, and used in the time-of-kill experiment as “germlings.” For the time-of-kill experiment, 0.5 × 10^5^ to 1 × 10^5^ cells/ml were prepared in 10 ml of RPMI 1640 liquid medium and incubated aerobically for 24 h at 30°C. Aliquots (20 μl) were removed at the indicated time points and serially diluted in RPMI 1640 liquid medium (in the absence of NP213 and in the presence of 3% [wt/vol] PASA to neutralize residual NP213), and 50 μl (in triplicate) was plated on PDA agar and incubated for up to 7 d at 30°C or until clear fungal colonies were evident in controls. The colonies were then counted to establish fungal killing.

### PI staining.

A spore suspension of T. rubrum NCPF0118 (1 × 10^4^ spores/ml) was exposed to 0, 500, and 1,000 mg/liter NP213 for 18 h at 30°C. After this, the fungi were exposed to PI, as modified from the manufacturer’s instructions in a FungaLight CFDA, AM/propidium iodide yeast vitality kit (Fisher Scientific, UK), where fungi were exposed to 16 μM PI for 15 min at 30°C. The cells were viewed by fluorescence microscopy (×400 magnification; Zeiss Axiovert 40), and the percentage of cells stained with PI was determined in six independent fields of view in duplicate experiments.

### SEM and TEM.

T. rubrum NCPF0118 was prepared for electron microscopy by growing in 12-well polystyrene tissue culture plates (Greiner Bio-one, UK) on sterile 0.2-μm-pore-size polycarbonate membrane filters (Thermo-Fisher Scientific) submerged in RPMI 1640 medium for 7 d at 30°C. After 7 days, fungi were exposed to either NP213 (2,000 mg/liter), TRB (0.01 mg/liter), or ciclopirox olamine (2 mg/liter) for 48 h at 30°C. Filters were then aseptically transferred to sterile Bijou containers (7 ml), submerged in 2.5% (vol/vol) glutaraldehyde in 100 mM sodium phosphate buffer (pH 7.3), and stored at 2 to 8°C for 48 h prior to processing for electron microscopy analysis. For SEM analysis, the cells were processed by ethanol and hexamethyldisilazane dehydration series before gold mounting. For TEM analysis, the cells were dehydrated by passing through ethanol and acetone series before being embedded in wax resin, stained with uranyl acetate/lead citrate stains to improve contrast, sectioned at 90 nm, and mounted onto copper grids for TEM analysis. TEM and SEM micrographs were acquired using a JEM-1400 Plus JEOL TEM and a Zeiss EVO MA10 SEM, respectively. Microscopy was performed in the Microscopy and Histology Core Facility at the University of Aberdeen (UK).

### Conventional determination of MIC and MFC values.

Antifungal susceptibility testing to determine the MIC was conducted by the broth microdilution procedure for filamentous fungi (M38-A2) (13). The MFC was determined according to the method described by Ghannoum et al. (56). Briefly, once MIC testing was completed, the contents of the wells were mixed thoroughly; 100 μl of culture from each well was spread plated onto PDA agar and incubated at 30°C for up to 7 days. The absence of any growth was deemed fungicidal, whereas growth from wells that contained concentration of antifungals at or above the MIC was deemed fungistatic. Experiments were carried out in triplicate with triplicate samples in each experiment. Selected HDPs have been shown to bind to polystyrene, resulting in reduced activity in antimicrobial susceptibility tests employing such plasticware (57). However, parallel MIC determinations using polypropylene and polystyrene microwell plates with T. rubrum NCPF0118 demonstrated no evidence of such an effect with NP213, with identical MICs in both polypropylene and polystyrene 96-well plates (data not shown). Since polystyrene 96-well plates are optically clear and give lower background optical densities at 530 nm than polypropylene plates, these were used in all relevant experiments.

### Modified antifungal susceptibility testing procedure.

In selected experiments, antifungal susceptibility testing was carried out as described above, but with the following modifications. RPMI 1640 liquid medium was substituted for 10 mM sodium phosphate buffer (pH 7.0) containing 0.1, 0.25, 0.5, 0.75, or 1.0% (wt/vol) powdered toenail suspensions and 0.0025% (wt/vol) alamarBlue (Thermo-Fisher Scientific). alamarBlue is a cell viability reagent that uses the reducing power of living cells to quantitatively measure viability that can be detected using a fluorescence-based plate reader. Human nails were obtained from an NHS podiatrist (NHS Grampian, UK), after obtaining appropriate ethical approval (NHS Grampian Research Ethics Committee REC reference 05/S0801/115) and informed written consent, from donors following nail avulsion performed because of ingrown toenails. All nails were disease-free, based on the podiatrist’s diagnosis. Nails were cut into small fragments (<5 mm) and ground to a fine powder in liquid nitrogen using a mortar and pestle. Following this, the powdered nails were passed through a fine-meshed sieve, and the sieved nail powder was used to prepare suspensions in 20 mM sodium phosphate buffer (pH 7.0), which was subsequently diluted 1:1 after addition of the fungal inoculum. Nail powder suspensions were sterilized by autoclaving for 20 min at 121°C.

Fungal metabolic activity was monitored by fluorescence (λex, 530 nm; λem, 590 nm) following the reduction of alamarBlue every 24 h for up to 168 h or until high metabolic activity (≥100,000 U) was observed in the inoculated controls in the absence of any antifungal agent using a BioTek Synergy HTX Multi-Mode Reader (BioTek U.S., Winooski, VT). As a control, antimicrobial susceptibility testing was conducted in RPMI 1640 liquid medium, and MICs were determined by measuring both the optical density and metabolic activity. Sodium phosphate buffer (pH 7.0) alone did not support dermatophyte growth (data not shown). The experiments were performed in triplicate with three replicates per each experiment.

### *In vitro* human nail model of *Trichophyton* infection and *ex vivo* treatment of onychomyotic nails.

An experimental model of *Trichophyton* spp. nail infection was optimized to simulate onychomycosis under laboratory conditions and to test the antifungal efficacy of NP213 and antifungal comparators (Fig. 5). As an inert support and to provide a moist atmosphere, sterile water agar plates were prepared in 90-mm petri dishes (1.5% [wt/vol] agar in sdH_2_O). Petri dishes were divided into three sections, and individual pieces of sterile silicone rubber tubing (1-cm diameter; 3-mm height) were placed in each section, allowing the investigation of three nail fragments per plate. This prevented direct contact between the nails and the agar surface or the lid of the petri dish. Such a setup was necessary because commercial agar preparation components may bind to cationic peptides, including defensins and polymyxins (58, 59), inhibiting their diffusion and reducing activity. Silicon tubing is permeable to oxygen and CO_2_ (60), and its use ensured O_2_ access to support fungal growth. Its diameter was sufficiently small to stably support the nail fragments.

Uninfected human nails (sourced under ethical approval, as described above in “Modified antifungal susceptibility testing procedure”) were cleaned of all residual skin and debris before cutting to size (ca. four fragments were obtained from an adult male great toe nail), and their surface area and thickness were determined using a micrometer (Fig. 5Bi). Nail fragments were submerged in 10 ml of dH_2_O and sterilized by autoclaving at 134°C for 20 min. The samples were then mounted with the ventral (nail bed side, concave) surface facing upward on the silicon tubing rings. Nail samples were allocated to provide nails of similar average size and thickness for each treatment. Nails were left to dry and incubated to ensure sterility for 7 days in a humid atmosphere at 30°C. Each nail fragment was inoculated on its ventral surface with a spore suspension containing ca. 2 × 10^7^ spores of *Trichophyton* spp. in 0.15 M NaCl (0.01 ml/cm^2^ nail surface area). Petri dishes were incubated at 30°C inside a sealed plastic box containing an sdH_2_O reservoir to avoid dehydration, for 14 days, at which point the hyphal growth was clearly visible on the entire surface of the nail fragment (Fig. 5Bii). Nails within the same petri dish were always exposed to the same fungal inoculum and treatment, mitigating the risk of cross-contamination. Randomly sampled nails were subjected to TEM analysis (described above) to confirm infection had been established with fungal penetration of the nail fragments (Fig. 5Biii, uninfected, and 5Biv, infected).

For *ex vivo* assessment of the antifungal activity of NP213, nails from patients diagnosed with onychomycosis (obtained under ethical approval as described above in “Modified antifungal susceptibility testing procedure”) were surface cleaned with 70% isopropyl alcohol swabs (Robinson Healthcare, Ltd., UK) but not sterilized.

All nail fragments, whether naturally infected or infected *in vitro*, were treated by application of NP213, comparator antifungal agents, or controls to the dorsal side of the nail. The dorsal (convex) aspect of the nail is that normally exposed to the environment. Antifungal agents were applied (0.01 ml/cm^2^ nail surface area) daily and distributed over the surface of the nail area with a sterile nail lacquer brush applicator (VWR International, Ltd., UK), as specified for each experiment.

After 28 days of daily treatment with NP213, nail fragments were transferred to sterile 2.0-ml microcentrifuge tubes containing 1 ml of SAB broth containing 6% (wt/vol) PASA to neutralize the residual NP213 (Fig. 3) (61) and with 100 mg of sterile 0.5-mm-diameter glass beads. For nail fragments treated with ciclopirox, SAB broth was supplemented with 800 μM iron(III) chloride to inactivate ciclopirox (62), a concentration that did not affect T. rubrum growth (data not shown). All other nails were processed in SAB broth with 100 mg of sterile 0.5-mm glass beads. The contents of the tubes were mixed thoroughly for 1 min using a bead-beater to release fungal cells from the nail matrix for enumeration, and stepwise 10-fold dilutions were prepared in SAB broth containing 3% (wt/vol) PASA. Samples were plated on PDA and incubated at 30°C for 14 days, and the colonies were counted. Recovery of microbes from nails from onychomycosis patients, both NP213 treated and untreated, was carried out using DTM agar (Becton Dickinson UK) rather than PDA.

### Statistical analysis.

All statistical analyses were carried out using Prism software (GraphPad, La Jolla, CA). Statistical significance was determined as described in the text.

## Supplementary Material

Supplemental file 1

Supplemental file 2
