# Supplementary material for: Improved Methods for Assessing Therapeutic Potential of Antifungal Agents against Dermatophytes and Their Application in the Development of NP213, a Novel Onychomycosis Therapy Candidate
Source: Antimicrob Agents Chemother. 2019 Apr 25;63(5):e02117-18. doi: 10.1128/AAC.02117-18 (PMC6496068; doi:10.1128/AAC.02117-18)
Supplement: Supplemental file 2 [file AAC.02117-18-s0002.pdf]

SUPPLEMENTARY MATERIAL

SUPPLEMENTARY TABLES

Table - MIC\Table S - NP213 MIC data.xlsx

TABLE S1 Minimum inhibitory concentration (MIC; mg/L) and minimum fungicidal concentration (MFC; mg/L) of NP213 versus dermatophytes (n = 117) using CLSI broth microdilution procedure (1) MICs are the median MIC, based on at least 3 biological replicates, each including 3 technical replicates.

SUPPLEMENTARY FIGURES

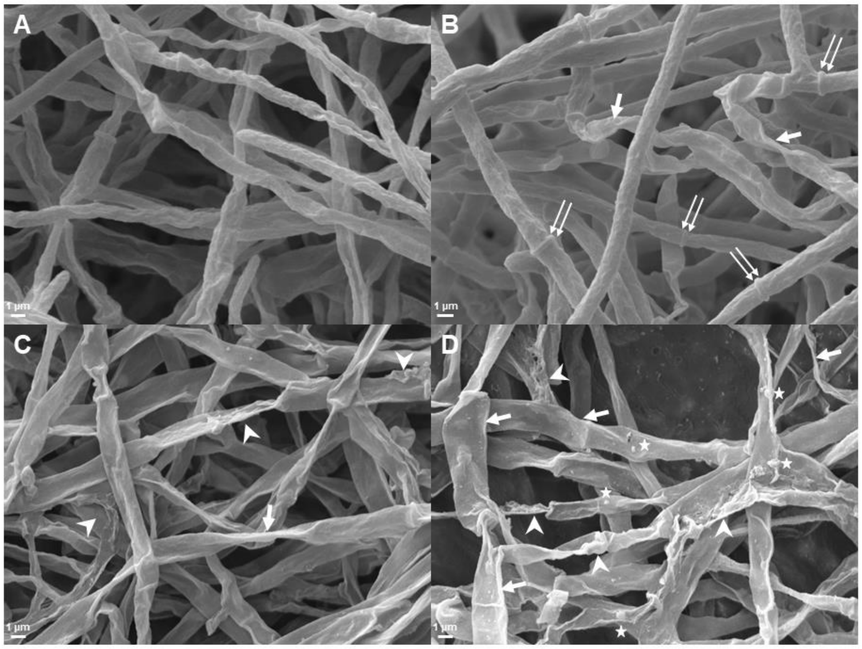

FIGURE S1 SEM analysis of the effect of exposure to increasing concentrations of NP213 on the morphology of *T. rubrum* NCPF0118; (A) 0 mg/ml NP213; (B) 1 mg/ml NPP213; (C) 10 mg/ml NP213; and (D) 100 mg/ml NP213. Single arrows represent flattening of the hyphae; double arrows represent bulges within the hyphae; arrowheads represent damage to the surface of the hyphae; asterisks represent possible blebbing on the hyphal surface. All sample treatments were conducted in triplicate and at least 30 images were acquired.

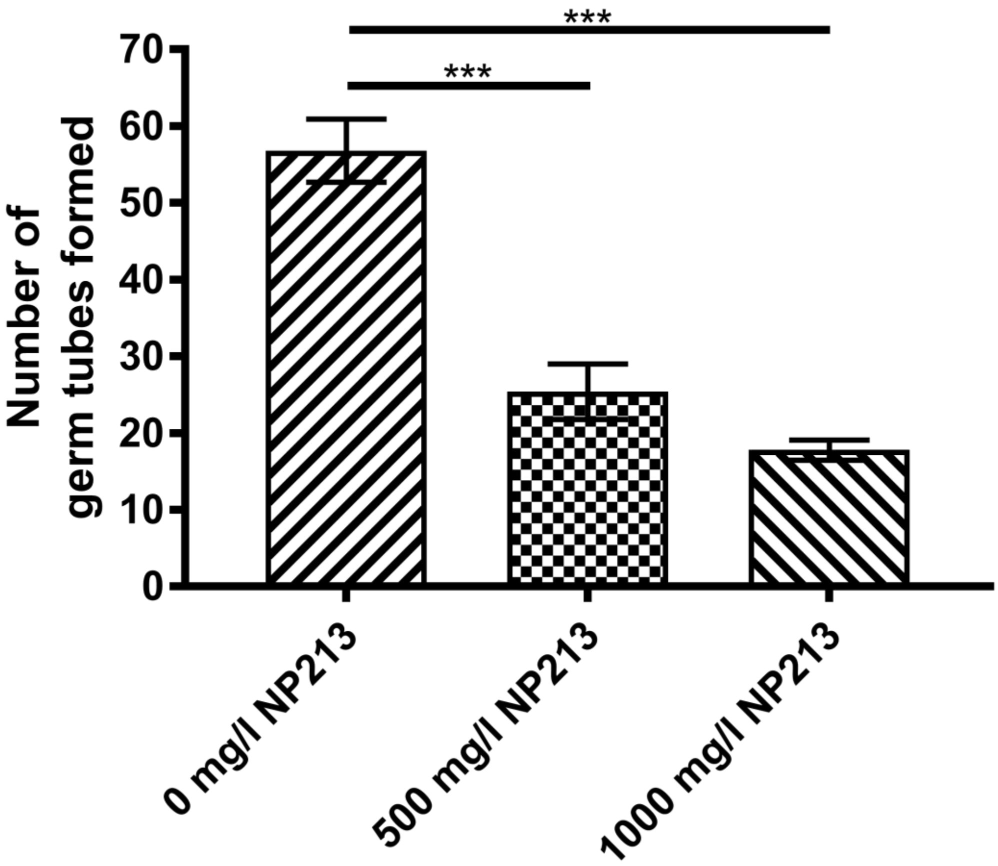

FIGURE S2 Effect of NP213 on fungal germ tube formation. Spore suspensions of *T. rubrum* NCPF0118 (1×10^4 spores) were incubated in RPMI-1640 liquid medium with either 0, 500, or 1000 mg/L NP213 (in triplicate) for 6 h at 30°C and analysed using phase contrast microscopy (× 400 magnification). The germ tubes formed in five different fields of view were counted for each sample. Significantly fewer germ tubes were formed (p<0.05) in spore suspensions exposed to 500 mg/L or 1000 mg/L NP213 than in unexposed spore suspensions, but the difference in the number of germ tubes formed from spores exposed to different NP213 concentrations was not significant (p>0.05, one-way ANOVA with Tukey's multiple comparisons test). Further, no significant differences in the length of the formed germ tubes were observed regardless of the presence or

absence of NP213 or different NP213 concentrations ($p>0.05$, one-way ANOVA with Tukey's multiple comparisons test; data not shown).

REFERENCES

1. CLSI. 2008a. Reference Method for Broth Dilution Antifungal Susceptibility Testing of Filamentous Fungi; Approved Standard - Second Edition (M38-A2). Clinical and Laboratory Standards Institute, Wayne, PA.
2. CLSI. 2008b. Reference Method for Broth Dilution Antifungal Susceptibility Testing of Yeasts; Approved Standard - Third Edition; Third Edition; M27-A3. Wayne, PA. Clinical and Laboratory Standards Institute.
3. Neron B, Menager H, Maufrais C, Joly N, Maupetit J, Letort S, Carrere S, Tuffery P, Letondal C. 2009. Mobyle: a new full web bioinformatics framework. Bioinformatics 25:3005-11.
